# High mortality in coronavirus disease 2019 (COVID-19)–suspect unit: Lessons learned for patient safety

**DOI:** 10.1017/ice.2021.91

**Published:** 2021-03-12

**Authors:** Anucha Apisarnthanarak, Surachai Chaononghin, Panipak Katawethiwong, David K. Warren

**Affiliations:** 1 Division of Infectious Diseases, Thammasat University Hospital, Pratum Thani, Thailand; 2 Division of Infectious Diseases, Washington University School of Medicine, Saint Louis, Missouri

*To the Editor*—The coronavirus disease 2019 (COVID-19) pandemic is a global healthcare emergency on a scale not seen in more than a century. With the emergence of new variants, COVID-19 is becoming potentially more contagious with transmission dynamics associated with intercontinental spread.^1–4^ To limit transmission of severe acute respiratory coronavirus virus 2 (SARS-CoV-2) in the hospital in Thailand, most hospitals have created special COVID-19–suspected units to care for all patients suspected to have COVID-19. At Thammasat University Hospital (Pratum Thani, Thailand), a COVID-19–suspect unit was created on February 1, 2020. This unit admits non–critically ill medical patients with special protocols (eg, specific laboratory procurement and respiratory sample collection protocol and management of patients by assigned personal for COVID-19) assigned at the initial sites of evaluation (eg, emergency department, outpatient department, emerging infectious diseases clinic) for patients admitted to the COVID-19–suspect unit. From February 1, 2020, to June 30, 2020, higher mortality was detected among patients who were admitted to this unit compared to patients admitted to regular medicine units [10 of 78 (12.8%) vs 46 of 678 (6.7%); *P* = .04], despite the comparable severity index between those units. The mean Charlson comorbidity index score of COVID-19–suspect unit was 2.2 (±1.7) and this score in regular medicine units was 2.4 (±1.9) (*P* = .56).

We performed a retrospective review of the patients who were admitted to a COVID-19–suspect unit from February through June 30, 2020, to evaluate potential reasons for the higher mortality in this unit. Data collected included patient demographics, underlying diseases, the initial evaluation site (eg, delay laboratory procurements, delay time to admission, and delay in critical medical measures such as intravenous fluid and antibiotic administration), final diagnoses, and causes of mortality. Analyses were performed using SPSS software, version 15 software (IBM, Armonk, NY). Categorical data were compared using the χ^2^ test or the Fisher exact test, as appropriate. We used the Mann-Whitney *U* test to compare continuous variables. Logistic regression was performed to assess predictors for mortality. Adjusted odd ratios (aORs) and 95% confidence intervals (CIs) were computed; a significant statistical difference was defined as *P* < .05.

During the study period, 1,060 patients were evaluated for COVID-19. Among these patients, 419 (39.5%) were suspected to have COVID-19 and were investigated, and 341 (81.3%) of these patients were managed as outpatients. In total, 78 (18.7%) of 419 patients were admitted to COVID-19 suspect unit. Of these 78 patients, 12 (15.3%) had hypertension, 10 (12.8%) had diabetes, and 8 (10.3%) had underlying pulmonary diseases. Notably, 12 patients (15.8%) had noninfectious diseases requiring special care (eg, gastrointestinal bleeding, acute coronary artery diseases, diabetic ketoacidosis, acute renal failure, acute asthma exacerbation). Of these 78 patients, 10 COVID-19–suspect inpatients (12.8%) died. A comparison of COVID-19 suspect inpatients who died versus those who survived is listed in Table 1. COVID-19 was confirmed in 17 patients (21.8%), and the causes for mortality included bacterial infections (8 of 10, 80%) and the noninfectious diseases diagnoses included diabetic ketoacidosis (1 of 10, 10%) and acute coronary artery diseases (1 of 10, 10%). Notably, lower mortality was detected among patients who were diagnosed with viral infections [0 of 10 (0%) vs 34 of 68 (50%); *P* = .004] and patients admitted from the emerging infectious diseases clinic [0 of 10 (0%) vs 29 of 68 (42.6%); *P* = .01] (Table 1). None of healthcare workers (HCWs) in this hospital became infected with SARS-CoV-2 during the study period.

**Table 1.** Comparison of 78 Patients Admitted to COVID-19 Suspect Unit, by In-Hospital Mortality

Variable	Total (n = 78)	Died (n = 10)	Survived (n = 68)	*P* Value
Age, median y (range)	40.5 (15–70.5)	55 (15–70.5)	37 (27–59)	0
Sex, female	36 (46)	6 (60)	32 (47.1)	0.74
**Underlying comorbidities**				
Hypertension	12 (15.4)	3 (25)	9 (13.2)	0.17
Diabetes	10 (12.8)	2 (20)	8 (11.8)	0.60
Lung disease	8 (10.3)	1 (10)	7 (10.3)	1
Heart disease	5 (6.4)	3 (30)	2 (2.9)	0.02
Kidney disease	3 (3.8)	0 (0)	3 (4.4)	1
**Initial evaluation site**				
Emergency department	40 (51.3)	10 (100)	30 (44)	0.001
Emerging infectious diseases unit	29 (37.2)	0 (0)	29 (42.6)	0.01
Outpatient department	9 (11.5)	0 (0)	9 (13.2)	0.59
**Delay processes of care**				
Laboratory procurement^a^	28 (29.5)	6 (60)	22 (32)	0.09
Time to admission^b^	49 (39.7)	5 (50)	44 (65)	0.36
Critical clinical management^c^	4 (5.1)	4 (40)	0 (0)	<.001
**Final diagnosis**				
Infectious diseases				
Viral infection^d^	34 (43.6)	0 (0)	34 (50)	0.004
Bacterial infections	29 (37.2)	9 (90)	20 (29.4)	<.001
Fungal infections	4 (5.1)	0 (0)	4 (5.9)	0.57
Noninfectious diseases^e^	12 (15.4)	2 (20)	10 (14.7)	0.19

Note. PCR, polymerase chain reaction.

a Defined as delay in obtaining blood culture and viral panel PCR for >60 min after ordered.

b Defined as delay in admission time >60 min from emergency department or >120 min from outpatient departments to COVID-19 suspect unit.

c Defined as aggressive fluid resuscitation, administration of antibiotics, blood transfusion, oxygen support and blood glucose control >60 min after ordered.

d Includes COVID-19 (17/78; 21.8), respiratory viral infection (18/78; 21.1%) and influenza 1 (1/78; 1.3%).

e Includes diabetic ketoacidosis, kidney failure, acute coronary artery disease, upper gastrointestinal bleeding, acute heart failure, acute asthma exacerbation, active systemic lupus erythematosus.

By multivariable analysis, a final diagnosis of bacterial infection (aOR, 13.7; 95% confidence interval [CI], 1.45–89.5; *P* < .001), initial evaluation in the emergency department (aOR, 10.8; 95% CI = 3.6–59.5; *P* = .001), and delayed time to admission (>60 minutes from emergency department or >120 minutes from outpatient departments) were associated with mortality in this unit (aOR, 7.7; 95% CI, 2.44–69.7; *P* = .005). Several processes of care identified as issues among patients admitted to the unit included delays in laboratory procurements (23 of 78, 29.5%), time to admission (49 of 78, 39.7%), and deployment of critical medical measures such as IV fluid and antibiotic administration (4 of 78, 5.1%).

We report a high mortality rate in a COVID-19–suspect unit in a Thai hospital. This mortality rate was 2 times higher than that of medical patients with comparable severity of illness admitted during the same period. This difference was related to several suboptimal processes in the care of patients requiring specialized medical care (eg, acute coronary artery disease, diabetic ketoacidosis, bacterial infections). In a previous report from Thailand, HCWs were overwhelmed with fear and anxiety regarding COVID-19.^5^ Such emotions affect patient care when HCWs are not willing to accept new patients or see admitted patients during epidemics, which may compromise patient safety.^5^ HCWs may be swayed by anecdotal stories that may impair clinical decision making. Anxiety and fear of contagion, despite the evidence of the effectiveness of personal protective equipment, may alter care.^5^

Despite the limitations of sample size and retrospective design, our study calls for a better emerging infectious disease preparedness plans in hospitals to incorporate the care for patients admitted to the COVID-19–suspect unit who may need special care. They should receive care without delay at the initial evaluation site, particularly the emergency department, before transfer to the COVID-19–suspect unit. Mechanisms for monitoring the processes of care among these patients are critical for their survival. Additional studies to evaluate strategies to improve the quality of care, as well as patient safety during epidemics, are needed.
